# Pathophysiology and Management of Chest Wall Pain after Surgical and Non-Surgical Local Therapies for Lung Cancer

**DOI:** 10.1016/j.jtocrr.2024.100690

**Published:** 2024-05-16

**Authors:** John Nikitas, Jane Yanagawa, Sandra Sacks, Edward K. Hui, Alan Lee, Jie Deng, Fereidoun Abtin, Robert Suh, Jay M. Lee, Paul Toste, Bryan M. Burt, Sha’Shonda L. Revels, Robert B. Cameron, Drew Moghanaki

**Affiliations:** a Department of Radiation Oncology, University of California, Los Angeles, Los Angeles, California; b Division of Thoracic Surgery, University of California, Los Angeles, Los Angeles, California; c Department of Anesthesiology, University of California, Los Angeles, Los Angeles, California; d Center for East-West Medicine, University of California, Los Angeles, Los Angeles, California; e Thoracic Imaging and Intervention, Department of Radiological Sciences, University of California, Los Angeles, Los Angeles, California; f Radiation Oncology Service, Greater Los Angeles Veterans Affairs Healthcare System, Los Angeles, California

**Keywords:** Chest wall pain, Lung cancer, Lung surgery, Image guided thermal ablation, Radiation therapy, Thoracotomy

## Abstract

Chest wall pain syndromes can emerge following local therapies for lung cancer and can adversely affect patients’ quality-of-life. This can occur after lung surgery, radiation therapy, or percutaneous image-guided thermal ablation. This review describes the multifactorial pathophysiology of chest wall pain syndromes that develop following surgical and non-surgical local therapies for lung cancer and summarizes evidence-based management strategies for inflammatory, neuropathic, myofascial, and osseous pain. It discusses a step-wise approach to treating chest wall pain that begins with non-opioid oral analgesics and includes additional pharmacologic treatments as clinically indicated, such as anticonvulsants, serotonin and norepinephrine reuptake inhibitors, tricyclic antidepressants, and various topical treatments. For myofascial pain, physical medicine techniques, such as acupuncture, trigger point injections, deep tissue massage, and intercostal myofascial release can also offer pain relief. For severe or refractory cases, opioid analgesics, intercostal nerve blocks, or intercostal nerve ablations may be indicated. Fortunately, palliation of treatment-related chest wall pain syndromes can be managed by most clinical providers, regardless of the type of local therapy utilized for a patient’s lung cancer treatment. In cases where a patient’s pain fails to respond to initial medical management, clinicians can consider referring to a pain specialist who can tailor a more specific pharmacologic approach or perform a procedural intervention to relieve pain.

## Introduction

Lung cancer remains the leading cause of cancer death.^1^ With increased early-detection screening efforts, more diagnosed lung cancers are early-stage and therefore amenable to local therapies, including surgery, radiation therapy, and percutaneous image-guided thermal ablation (IGTA).^2^ With appropriate therapy for early-stage lung cancer, 10-year survival rates can exceed 70%.^3^ Yet, these local therapies can be associated with treatment-related chest wall pain that can adversely impact patients’ quality-of-life for extended periods of time.

The etiology of chest wall pain is associated with the type of local therapy delivered. For example, chest wall pain that occurs following surgery is generally due to trauma from the incision and surgical manipulation of the chest wall.^4^ It occurs following radiation therapy due to ischemic, necrotic, and fibrotic changes in the irradiated tissue.^5^ It occurs following IGTA due to trauma of the surrounding tissue from needle insertion, heating, or freezing.^6^

While the iatrogenic mechanisms leading to chest wall pain after surgery, radiation therapy, or IGTA may be unique, there are shared pathophysiological components including inflammatory, neuropathic, myofascial, and osseous injuries as described below.^7^

Inflammatory pain occurs due to the immune system’s response to acute tissue damage, leading to the release of cytokines, chemokines, and prostaglandins that act as pain mediators.^8^ This causes inflammatory hyperalgesia, where nerve terminals near the injured tissue become over sensitized. This type of pain persists until inflammation resolves and responds well to anti-inflammatory medication.^9^

Neuropathic pain arises from peripheral nerve damage, resulting in heightened neuronal activity and central sensitization. This alteration in pain processing causes persistent hyperalgesia (heightened sensitivity to painful stimuli) and allodynia (pathological sensitivity to stimuli that are not normally painful).^9^ Neuropathic pain can be differentiated from inflammatory pain by the presence of allodynia, by its persistent effects beyond the resolution of inflammation, by the poor response to anti-inflammatory medication, or by the presence of negative symptoms, including sensory or motor loss.^9^^,^^10^

Myofascial pain develops due to trauma inflicted on muscle fibers, leading to ischemia. This disrupts the function of intracellular calcium pumps, resulting in increased levels of intracellular calcium. Consequently, this triggers persistent muscle contractions, stiffness and tenderness of the chest wall, and formation of myofascial trigger points.^11^ Local stimulation of trigger points reproducing a patient’s pain is pathognomonic for myofascial pain.

Lastly, osseous pain occurs due to rib fractures in the treated chest wall during surgery or following radiation therapy or IGTA.^6^^,^^12^^,^^13^ This can be diagnosed via computed tomography (CT) scans, though fractures are not always immediately visible.

Due to its multifactorial nature, management of chest wall pain syndrome is complex and often benefits from a multimodal approach. This review intends to better elucidate the pathophysiology of chest wall pain syndromes that arise post-lung cancer treatment and summarize evidence-based approaches for their management. These strategies include non-opioid oral analgesics, neuropathic pain medications, topical treatments, opioid analgesics, physical medicine techniques, and procedural interventions.

## Pathophysiology of Chest Wall Pain after Thoracic Surgery

### Thoracotomy

Traditional lung resections involve an approximately six- to nine-inch posterolateral thoracotomy incision, typically between the fifth and sixth ribs. Depending on the location of the tumor and the specific surgical technique, the muscles overlying the chest wall are dissected and the ribs may be retracted to allow for better access to the lungs. During this process, traumatic injuries occur to the costochondral and costovertebral joints, ribs, muscles, pleural lining, and intercostal nerves (Table 1).^4^ This can lead to inflammatory pain until wound healing resolves, neuropathic pain due to transection of the intercostal and pleural nerves, myofascial injury and trigger point formation due to injury of the chest wall muscles, and rib fractures due to intraoperative retraction of the ribs.

**Table 1:** Causes of Chest Wall Pain Syndrome

Causes		Type of Pain	Pathophysiology
Surgery	Thoracotomy	Inflammatory	Post-operative tissue inflammation and wound healing.
	Minimally invasive surgery	Neuropathic	Intercostal or pleural nerve transection during surgery.
		Myofascial	Myofascial tissue disruption and trigger point formation due to surgical manipulation of the ribs and intercostal space.
		Osseous	Rib fracture during intraoperative retraction and manipulation of the ribs.
Radiation Therapy	Conventionally fractionated radiotherapy	Inflammatory	Post-radiation inflammation due to oxidative damage in the irradiated tissue.
	Stereotactic body radiotherapy	Neuropathic	Intercostal or pleural nerve compression due to fibrosis of nerve sheaths and normal tissue atrophy.
		Myofascial	Myonecrosis of the pectoralis muscle or soft tissue fibrosis in the chest wall.
		Osseous	Rib fracture due to injury of the Haversion canals, leading to ischemia and sclerosis of the bone marrow, decreased osteoblasts, and decreased collagen production.
Image-Guided Thermal Ablation	Radiofrequency ablation	Inflammatory	Post-procedural tissue inflammation and wound healing.
	Microwave ablation	Neuropathic	Intercostal or pleural nerve injury from probe placement or from overlap between the nerves and the ablation zone.
	Cryoablation	Myofascial	Myofascial tissue disruption or injury from probe placement or from overlap between the chest wall and the ablation zone.
		Osseous	Rib fracture following ablation from overlap between the ribs and the ablation zone.

Following a thoracotomy, approximately 30% to 40% of patients report pain beyond three months with 5% to 10% reporting a score greater than 5 on a 10-point scale.^10^ This can lead to prolonged opioid use beyond 90 days post-operatively in approximately one in four patients.^14^ Factors such as how the ribs were manipulated, how retraction was performed, whether muscles were split, and the number of inserted chest tubes affect the risk, severity, and duration of chest wall pain.^15^

### Minimally Invasive Surgery

In contrast to thoracotomy, video-assisted thorascopic surgery (VATS) is a minimally invasive surgical technique that uses small incisions to insert a surgical camera and thorascopic tools into the thorax. At least one incision site must be expanded to allow for removal of the resected lung tissue, which carries the highest risk of intercostal nerve injury.^16^ Multiple studies have demonstrated that VATS is associated with lower rates of postoperative pain, perioperative blood loss, hospitalization duration, and overall quality-of-life compared to thoractomy.^17^, ^18^, ^19^, ^20^ A 2016 Danish study by Bendixen et al.^17^ that randomized 206 patients between anterolateral thoracotomy and VATS found that in the first year following surgery, VATS led to significantly lower post-operative moderate-to-severe pain (defined as ≥3 on a 10-point scale, *p* < 0.0001) and significantly higher patient-reported quality-of-life (*p* = 0.014). In that report, 9% of patients experienced moderate-to-severe chest wall pain one year after VATS versus 14% after thoracotomy, suggesting that chest wall pain can still occur despite the less invasive approach.

## Pathophysiology of Chest Wall Pain after Non-Surgical Local Therapies

### Conventionally Fractionated Radiation Therapy

Conventionally fractionated radiation therapy (CFRT) is delivered to patients with locally advanced NSCLC with low doses of daily radiation (1.8–2.0 Gy/fraction) given over approximately six weeks with the goal of causing lethal DNA damage within tumor cells. It is often delivered concurrently with chemotherapy, which increases the likelihood of both tumor death and adjacent normal tissue injury. CFRT is an uncommon cause for chest wall pain, although it can occur with symptoms like those following surgery, with patients reporting pain, paresthesias, or hypoesthesias.^21^ The onset of symptoms is delayed, with most patients reporting the emergence of symptoms 10 to 22 months after CFRT.^22^

The pathophysiology of radiation-induced chest wall pain is multifactorial. It is believed to be caused by recruitment of inflammatory cells to the irradiated tissue following oxidative damage (Table 1).^23^ It is also believed to be a consequence of normal tissue atrophy and chronic ischemia contributing to peripheral nerve injury.^22^ Autopsy findings have shown marked fibrosis of the nerve sheaths with demyelination and atrophy within irradiated tissues.^22^ Fibrosis often results from chronic ischemia and can compress peripheral nerves.^5^ Chest wall pain may also be related to delayed rib fractures that can be invisible on chest X-ray and CT. This occurs due to injury of the Haversion canals, leading to reduced blood flow and sclerosis of the bone marrow, decreased osteoblasts, and decreased collagen production.^12^ Finally, radiation therapy can rarely cause myonecrosis of the pectoralis muscles, leading to myofascial pain.^24^

### Stereotactic Body Radiation Therapy

Stereotactic body radiation therapy (SBRT) delivers large doses of radiation to lung tumors in five fractions or less to improve the likelihood of local control versus CFRT.^25^^,^^26^ This is associated with higher rates of chest wall pain, with up to 18% of patients reporting chest wall pain after treatment.^27^^,^^28^ It occurs at a median of 7.1 months after SBRT and lasts for a median duration of 4.7 months.^5^ There is an association between chest wall pain syndrome after SBRT and short tumor-to-chest wall distance.^29^ The highest risk area is the posterolateral chest.^30^

### IGTA

Another non-surgical treatment for lung tumors is IGTA. In this procedure, a probe is inserted percutaneously through the intercostal space into the lung tumor under CT guidance to deliver radiofrequency ablation (RFA), microwave ablation (MWA), or cryoablation. This causes immediate tumor cell necrosis inside the ablation zone.^31^^,^^32^ Chest wall pain syndrome can occur after each IGTA modality due to traumatic or ablation-related injury of the intercostal nerves, nerves of the pleural lining, myofascial tissue, or ribs (Table 1).^6^ Chest wall pain is associated with proximity of the ablation zone to the chest wall.^33^ In a single-center study, Alexander et al.^6^ reported a 13.5% incidence of rib fractures in close proximity to ablation zones among 195 lung tumors treated with RFA and MWA. Chest wall pain is less common with cryoablation versus other forms of IGTA.^34^ RFA is most associated with chest wall pain, with one meta-analysis reporting a pooled incidence of 14.1% compared with 2% for MWA and as low as 0% for cryoablation.^35^, ^36^, ^37^ This is hypothesized to occur due to better preservation of the perineurium and epineurium following cryoablation, which facilitates nerve regeneration.^34^

## Evaluating Chest Wall Pain

Chest wall pain following local therapy for lung cancer has a broad differential diagnosis, which includes acute coronary syndromes, pulmonary embolism, tumor progression, pneumonia, abscess, and traumatic rib fracture in addition to treatment-related chest wall pain syndrome. As such, the management of patients with chest wall pain following lung cancer local therapies requires obtaining a detailed history, physical exam, labs, and imaging. Imaging should include a chest CT but can also include magnetic resonance imaging of the chest, which can identify signs of chest wall injury.^38^

## Management of Chest Wall Pain Syndrome

Chest wall pain occurring immediately after lung resection or IGTA is readily attributable to the procedure and is frequently managed by the treating team. However, chest wall pain developing weeks to months after surgery, CFRT, SBRT, or IGTA may present to clinicians in a primary care, emergency medicine, or other specialty setting. Often, palliation of treatment-related chest wall pain syndromes can be managed by most physicians regardless of the type of local therapy utilized for a patient’s lung cancer treatment (Table 2). As will be detailed, clinicians can begin with a step-wise approach that starts with non-opioid oral analgesics (non-steroidal anti-inflammatory drugs [NSAIDs] and acetaminophen), with escalation to other drug classes as indicated.^39^ If a patient’s pain fails to respond to this approach, clinicians can consider a referral to a pain management specialist who can tailor a more specific pharmacologic approach or perform a procedural intervention. In patients who have suspected myofascial pain, an early referral to physical therapy or integrative medicine can be considered.

**Table 2:** Treatments for Chest Wall Pain Syndrome

Intervention	Examples	Mechanism of Action	Use	Benefits	Risks
Non-Opioid Oral Analgesics	NSAIDs: ibuprofen and naproxen	Inhibit COX-1 and COX-2 enzymes	Any pain, especially if inflammatory pain	Analgesia and anti-inflammatory properties	Gastrointestinal ulcers, dyspepsia, reduced glomerular filtration rate, and increased cardiovascular events
	Acetaminophen	Positive effects on serotonergic descending inhibitory pathways	Any pain	Analgesia	Hepatotoxicity
Oral Glucocorticoids	Dexamethasone, prednisone, and prednisolone	Inhibit prostaglandin synthesis, reduce vascular permeability, and reduce spontaneous discharges from injured neurons	Inflammatory, neuropathic, or osseous pain	Anti-inflammatory, reduce tissue edema	Weight gain, insomnia, gastrointestinal ulcers, psychiatric disturbances, reduced bone mineralization, and increased infections
Anticonvulsants	Gabapentin and pregabalin	Bind the presynaptic voltage-gated calcium channels in the central nervous system	Neuropathic pain	Modulate dysfunctional nerve signaling	Dizziness, somnolence, ataxia, and confusion
Serotonin and Norepinephrine Reuptake Inhibitors	Duloxetine, venlafaxine, and desvenlafaxine	Block reuptake of both serotonin and norepinephrine	Neuropathic pain	Modulate dysfunctional nerve signaling	Nausea, xerostomia, and weight gain
Tricyclic Antidepressants	Amitriptyline	Block reuptake of serotonin and norepinephrine and competitively antagonize alpha cholinergic, muscarinic, and histaminergic receptors	Neuropathic pain	Modulate dysfunctional nerve signaling	Urinary retention, xerostomia, cardiac conduction abnormalities, and confusion
Topical Treatments	Lidocaine, baclofen, diclofenac, ketamine, and gabapentin	Varies depending on drug(s) used	Inflammatory or neuropathic pain	Vary depending on drug(s) used	Dermatitis
Opioid Analgesics	Tramadol, hydrocodone, oxycodone, and morphine	Activate opioid receptors in the central nervous system	Severe pain of any etiology, especially if osseous pain	Analgesia	Constipation, drowsiness, nausea, and dizziness
Physical Medicine Techniques	Acupuncture	Enhance endogenous opiates	Refractory pain of any etiology	Analgesia	Discomfort
	Trigger point injections	Disruption of trigger points via mechanical effect	Myofascial pain	Analgesia	Discomfort, skin discoloration, and allergic reaction
	Deep tissue massage therapy	Reflexive hyperemia	Myofascial pain	Relieve myofascial tension and improve fascial flexibility	Discomfort
	Intercostal release exercises	Reflexive hyperemia	Myofascial pain	Relieve myofascial tension and improve fascial flexibility	Discomfort
Intercostal Nerve Ablation	Radiofrequency ablation and cryoablation	Block intercostal nerve signaling	Severe pain of any etiology	Long-term analgesia	Discomfort, pneumothorax, hemorrhage, and pain re-emergence

COX, cyclooxygenase; NSAIDs, non-steroidal anti-inflammatory drugs.

## Pharmacologic Management

### Non-Opioid Oral Analgesics

NSAIDs inhibit COX-1 and COX-2 enzymes to address the inflammatory component of chest wall pain.^40^ Acetaminophen reduces pain via positive effects on the serotonergic descending inhibitory pathways in addition to the opioid, eicosanoid, and nitric oxide-containing pathways.^41^ An analysis of three randomized controlled trials (RCTs) involving 1647 participants demonstrated that the combination of acetaminophen and NSAIDs offers superior analgesia compared to NSAIDs alone.^42^ It is important to recognize long-term NSAID use is associated with gastrointestinal ulcers, dyspepsia, acute or chronic kidney disease, and increased cardiovascular events.^43^ While acetaminophen is associated with fewer adverse effects, hepatoxicity can occur when daily doses exceed 4000 mg.^44^ When the combination of NSAIDs and acetaminophen does not address all pathophysiological components of chest wall pain, intensification of medical therapy may be required.

### Oral Glucocorticoids

Oral glucocorticoids such as dexamethasone, prednisone, and methylprednisolone also play a role in treating chest wall pain syndrome.^45^ They address inflammatory pain by reducing prostaglandin synthesis, vascular permeability, and tissue edema; they address neuropathic pain by reducing spontaneous discharges from injured neurons, and they improve osseous pain.^45^^,^^46^ However, glucocorticoids are associated with increased appetite and weight gain, proximal muscle weakness, insomnia, gastrointestinal ulcers, psychiatric disturbances, and reduced bone mineralization.^45^^,^^46^ Glucocorticoids are also associated with an increased risk of infections, especially in patients who have recently received chemotherapy and may be immunosuppressed.^47^ Glucocorticoids are generally given at the lowest effective dose and limited to only a few weeks. Glucocorticoids should be avoided in patients who are still healing from recent surgery and are advised to be administered with proton pump inhibitors or sulfamethoxazole and trimethoprim prophylaxis as clinically appropriate. Physicians who practice in areas with high incidence of *M. tuberculosis* should consider screening patients for tuberculosis prior to initiating glucocorticoids.

### Anticonvulsants

Gabapentin is an anticonvulsant drug that can be considered for patients with neuropathic pain refractory to non-opioid oral analgesics.^48^ Gabapentin reduces pain by inhibiting the upregulation of α2δ-1 subunits that occurs following nerve injury to the dorsal horn neurons.^49^ Sihoe et al.^50^ used gabapentin to treat 45 patients with persistent chest wall pain following thoracic surgery or trauma. Patients were titrated up to 300 mg three times daily, leading to 73% of patients reporting an improvement in pain with 42% reporting greater than 50% reduction in 10-point pain score. Stephans et al.^39^ examined 9 patients with SBRT-related pain treated with gabapentin, demonstrating that it helped some of the more severe cases of toxicity. Gabapentin is well-tolerated with less than 4% of patients discontinuing treatment due to an adverse effect.^51^ However, adverse effects can occur and include symptoms of dizziness, somnolence, ataxia, and confusion. Adverse effects are more likely to occur at high doses (≥1800 mg/day) or in patients with severe renal impairment.^49^

Other anticonvulsants that have been studied for neuropathic pain include pregabalin, topiramate, zonisamide, carbamazepine, and oxcarbazepine. Of these, pregabalin has the best evidence supporting its efficacy.^51^ Among patients who received chemoradiation for lung cancer and developed neuropathic chest wall pain, pregabalin (150 mg twice daily for 3 mo) was shown to significantly improve Leeds Assessment of Neuropathic Symptoms and Signs (7-item pain scale out of 24 total points) and visual analogue scale (visual marking by patients on a 10-cm line to represent severity of pain from 0 to 10 cm) scores at one month and three months (*p* < 0.001 for all). Only two out of 22 patients discontinued treatment due to drug-related adverse effects.^52^ Other anticonvulsants lack strong evidence to support their use in pain management and had significantly higher rates of adverse effects like dizziness, drowsiness, or confusion compared to gabapentin and pregabalin.^51^

### Serotonin and Norepinephrine Reuptake Inhibitors

Serotonin and norepinephrine reuptake inhibitors (SNRIs) can also be considered for neuropathic chest wall pain refractory to non-opioid oral analgesic medication. SNRIs include duloxetine, venlafaxine, and desvenlafaxine. They block reuptake of both serotonin and norepinephrine, resulting in increased neurotransmitter concentrations in the synaptic cleft. Several meta-analyses have shown that duloxetine and venlafaxine significantly reduce neuropathic pain levels compared to placebo without significant toxicity rates.^51^^,^^53^^,^^54^ Among 22 patients who received chemoradiation for lung cancer and developed neuropathic chest wall pain, duloxetine (60 mg daily) was shown to significantly improve Leeds Assessment of Neuropathic Symptoms and Signs and visual analogue scale scores at one month and three months (*p* < 0.001 for all).^52^ No patients had to discontinue treatment due to drug-related adverse effects. Adverse effects can occur with SNRIs and include nausea, xerostomia, and weight gain.^51^

### Tricyclic Antidepressants

Tricyclic antidepressants (TCAs) are a less commonly used oral medication class that can be considered for neuropathic chest wall pain refractory to non-opioid oral analgesic medications, which include amitriptyline, nortriptyline, clomipramine, desipramine, and doxepin. They block reuptake of serotonin and norepinephrine, while also serving as competitive antagonists to alpha cholinergic, muscarinic, and histaminergic receptors. Amitriptyline is the most studied TCA for treating neuropathic pain.^51^ Unfortunately, there are no specific trials that used TCAs to treat chest wall pain syndrome. The most robust evidence available is a meta-analysis of patients with neuropathic pain, which included post-surgical and cancer-related pain. According to the meta-analysis, among the 18 RCTs that studied TCAs for neuropathic pain, rates of patients reporting a reduction of 50% or more in their pain levels were 28% higher in the TCA arms compared to the placebo arms. Most of these trials used amitriptyline in the TCA arm. Reported adverse effects included urinary retention, xerostomia, cardiac conduction abnormalities, and confusion.^51^

### Topical Medications

In addition to oral medications, there are topical medications that can treat neuropathic pain with fewer adverse effects. These can include ketamine, anesthetics (lidocaine), muscle relaxants (baclofen or cyclobenzaprine), and NSAIDs (ketoprofen or diclofenac). Ketamine acts as an NMDA receptor antagonist, GABA_A_ receptor agonist, and opioid receptor re-sensitizer. It has been shown to help with neuropathic pain both centrally and peripherally. When applied topically, it offers significant pain relief without significant systemic absorption or clinically reported side effects.^55^ Lidocaine inhibits voltage-gated sodium channels, temporarily blocking nerve signaling. A systematic review of neuropathic pain treatments by Wolff et al.^56^ found that topical lidocaine was comparable in efficacy to oral medications like amitriptyline, pregabalin, and gabapentin but with fewer adverse effects. A systematic review by Maloney et al.^57^ similarly found that topical lidocaine can be highly effective for neuropathic pain syndromes while maintaining an excellent safety profile. Topical NSAIDs can also provide analgesia with lower systemic side effects than oral NSAIDs, including lower rates of cardiovascular events and less of an effect on platelet function.^57^ However, topical NSAIDs have not been well-studied with RCTs in the setting of neuropathic pain.

### Opioid Analgesics

In cases of severe chest wall pain refractory to other treatment modalities, especially when a symptomatic rib fracture has been identified, opioid analgesics may offer short-term relief. Opioids activate opioid receptors in the central nervous system to block pain signals and increase dopamine levels. Examples of common opioids prescribed in the outpatient setting include tramadol, hydrocodone, oxycodone, and morphine. While opioids can be highly effective for managing acute pain, such as in the case of a rib fracture, they have more limited efficacy for neuropathic pain compared to placebo (response rate of 47% versus 30%).^58^ Opioid use is associated with several adverse effects, including constipation, drowsiness, nausea, and dizziness and can cause fatal overdoses.^58^ There is also a significant risk of developing tolerance and dependence to opioids from long-term use.^59^^,^^60^ Therefore, opioids should be carefully managed and administered in conjunction with non-opioid interventions.^60^

### Pentoxifylline and Vitamin E

In cases of chest wall pain following CFRT or SBRT, trials of pentoxifylline and vitamin E have been reported to help normal tissue recovery from late effects of radiation therapy. This is best reported in cases of osteoradionecrosis of the ribs.^61^ Pentoxifylline works as a non-selective phosphodiesterase inhibitor and decreases blood viscosity by increasing red blood cell flexibility. This improves blood flow and oxygenation in irradiated, fibrotic tissue. Vitamin E is an antioxidant that protects cells against free radicals, including those produced from radiation therapy. Vitamin E has anti-inflammatory and anti-fibrotic effects.^62^ Both medications can cause mild gastrointestinal side effects and should be avoided in combination with medications like warfarin due to an increased risk of bleeding.

## Physical Medical Techniques

Physical medicine techniques can be considered as alternatives or adjuncts to analgesic medication in all patients with chest wall pain who do not experience satisfactory relief from pharmacologic management, especially when myofascial pain is suspected. These techniques include acupuncture, trigger point injections, deep massage therapy, and intercostal release exercises.

### Acupuncture

Acupuncture entails inserting small needles into specific acupuncture points to provide pain relief to patients by enhancing endogenous opiates.^63^ According to a meta-analysis of 39 RCTs studying acupuncture in the setting of osteoarthritis, shoulder pain, or nonspecific musculoskeletal pain, acupuncture offered superior pain relief compared to sham acupuncture and no acupuncture (*p* < 0.001 for both).^64^ The benefits of acupuncture persisted over time with only a 15% decrease in efficacy after one year of consistent use. Another study by Roshanzamir et al.^65^ enrolled 30 patients shortly after undergoing open-heart surgery. Patients were randomized 1:1 between receiving and not receiving acupuncture to their bilateral acupoints in addition to standard-of-care exercise therapy and routine analgesia. They found that patients randomized to acupuncture had higher rates of improvement in their pain scores (*p* < 0.001) and lower analgesic use (*p* < 0.001).

### Trigger Point Injections

An alternative approach to treat myofascial pain is trigger point injections. Providers trained in this technique identify myofascial trigger points where patients have localized tenderness and a referred pain pattern and inject them with either local anesthetics (lidocaine, bupivacaine, or prilocaine), a mixture of local anesthetic and glucocorticoids (e.g., lidocaine with triamcinolone acetonide), botulinum toxin type A, or normal saline.^66^ The literature remains inconsistent regarding which injection solution is most effective.^66^ An RCT of 28 patients with myofascial pain randomized patients to injections with normal saline versus lidocaine with triamcinolone acetonide.^67^ They found that patients in both arms had a three-point improvement in their pain levels on a 10-point scale after two weeks, and that there was no significant difference between these two interventions. Many clinicians are therefore favoring the use of normal saline due to the reduced risk of hypersensitivity reactions, drug allergies, or local anesthetic toxicity.

### Deep Tissue Massage Therapy

A less invasive therapy to address myofascial chest wall pain is deep tissue massage therapy with muscle stripping.^68^ This technique is performed by physical therapists to cause reflexive hyperemia and improve muscle fascia flexibility. A retrospective review by Hsu et al.^68^ found that muscle stripping in patients following thoracoscopic surgery led to significantly lower pain scores, shorter hospitalization duration, and reduced need for additional analgesics compared to conventional analgesia alone (*p* < 0.001 for all).

### Intercostal Myofascial Release Exercises

An alternative technique used by physical therapists to treat myofascial pain is performing intercostal myofascial release exercises. “Bucket handle release” and “surfing the ribs” techniques are used on the intercostal muscles to relieve myofascial tension and improve patient comfort with respiration. This has not been specifically studied for chest wall pain syndrome. However, a meta-analysis of myofascial release exercises for conditions like fibromyalgia, cervical spine pain, and lumbar spine pain found encouraging results among nine RCTs. They showed that myofascial release exercises had superior pain relief compared to sham treatment or no treatment.^69^

## Intercostal Nerve Block and Nerve Ablation

For patients with severe chest wall pain that is refractory to medical management, a referral to a pain specialist can be considered for intercostal nerve blocks, which can temporarily relieve symptoms. In this approach, palpation or image-guidance (fluoroscopy, ultrasound, or CT) are used to directly inject intercostal nerves with approximately 2 mL of 0.25% to 0.5% bupivacaine per intercostal nerve using 23- to 25-gauge needles.^70^^,^^71^ Glucocorticoids can be used in conjunction with local anesthetic.^72^ Intercostal nerve block are used to both confirm the site and source of the pain and to provide pain relief. If the duration of pain relief is satisfactory, repeat temporary nerve blocks can be performed before nerve ablation, or neurolysis, is undertaken. Often, a prolonged anesthetic effect occurs with partial or complete alleviation of pain.

For longer-lasting effect, intercostal nerve ablation can be performed under CT or ultrasound-guidance using RFA or cryoablation as a form of semi-permanent blockade. In RFA, medium-frequency alternating current is used to induce coagulation necrosis and nerve cell death.^71^ In cryoablation, a cryoprobe is used to freeze the target and disrupt axonal continuity between the sensory nerve endings and the central nervous system.^34^ In both cases, local anesthetic is injected before proceeding with ablation.^73^ The target is the nerve ramus that forms the intercostal nerve approximately 1 cm lateral to the intervertebral foramina. These interventions are most effective in patients who have pain localized to a few dermatome levels, such as in cases of pain related to a surgical incision or ablation site.

Both RFA and cryoablation have promising data. In a case series of two patients with post-surgical intercostal neuralgias, RFA led to a significant improvement in patients’ pain with no procedural complications.^74^ In a retrospective series of 18 patients who underwent cryoablation for post-thoracotomy pain syndrome, the authors reported a mean improvement in pain scores of 3.4 points on a 10-point scale following the procedure.^75^ No procedural complications were reported. Cryoablation does not typically have a permanent effect on nerves since the perineurium and epineurium are very fibrous tissues and can resist damage from freezing.^34^ Therefore, over the subsequent weeks to months, the perineurium and epineurium can facilitate regrowth of the nerve and pain re-emergence, requiring retreatment. In one study of 13 patients with post-thoracotomy pain who were treated with cryoablation, two patients experienced pain re-emergence that required retreatment at 6 and 10 months.^4^ In contrast, RFA causes more permanent destruction of myelinated fibers. Nonetheless, a similar nerve regeneration phenomenon has been reported with RFA where macrophage migration and Schwann cell proliferation can lead to axonal sprouting and nerve regeneration.^72^ Overall, cryoablation is the preferred modality for intercostal nerve ablation.

## Conclusion

Chest wall pain after surgical and non-surgical therapies for lung cancer has overlapping etiologies and management strategies. The underlying pathophysiology of the pain is multifactorial in nature and involves inflammatory, neuropathic, myofascial, and osseous components. In most instances, the duration of pain is self-limited. For patients with persistent or debilitating chest wall pain syndromes, management strategies can include analgesic medications, topical treatments, physical medicine techniques, and intercostal nerve block and ablation. Fortunately, palliation of treatment-related chest wall pain syndromes can be managed by most clinical providers, regardless of the type of local therapy utilized for a patient’s lung cancer treatment. When medical management fails, referring to pain specialists is recommended.

## CRediT Authorship Contribution Statement

**John Nikitas:** Conceptualization, Methodology, Investigation, Writing-original draft.

**Jane Yanagawa:** Writing - review and editing.

**Sandra Sacks:** Writing - review and editing.

**Edward K. Hui:** Writing - review and editing.

**Alan Lee:** Writing - review and editing.

**Jie Deng:** Writing - review and editing.

**Fereidoun Abtin:** Writing - review and editing.

**Robert Suh:** Writing - original draft, Writing - review and editing.

**Jay M. Lee:** Writing - review and editing.

**Paul Toste:** Writing - review and editing.

**Bryan M. Burt:** Writing - review and editing.

**Sha’Shonda L. Revels:** Writing - review and editing.

**Robert B. Cameron:** Writing - review and editing.

**Drew Moghanaki:** Conceptualization, Methodology, Writing - original draft, Writing - review and editing.

## Disclosure

The authors declare no conflict of interest.

## References

[1] Bray F., Ferlay J., Soerjomataram I., Siegel R.L., Torre L.A., Jemal A. (2018). Global cancer statistics 2018: GLOBOCAN estimates of incidence and mortality worldwide for 36 cancers in 185 countries. CA Cancer J Clin.

[2] Ganti A.K., Klein A.B., Cotarla I., Seal B., Chou E. (2021). Update of incidence, prevalence, survival, and initial treatment in patients with non-small cell lung cancer in the US. JAMA Oncol.

[3] Ito H., Suzuki K., Mizutani T. (2020). Long-term survival outcome after lobectomy in patients with clinical T1 N0 lung cancer. J Thorac Cardiovasc Surg.

[4] Yasin J., Thimmappa N., Kaifi J.T. (2020). CT-guided cryoablation for post-thoracotomy pain syndrome: a retrospective analysis. Diagn Interv Radiol.

[5] Dunlap N.E., Cai J., Biedermann G.B. (2010). Chest wall volume receiving >30 Gy predicts risk of severe pain and/or rib fracture after lung stereotactic body radiotherapy. Int J Radiat Oncol Biol Phys.

[6] Alexander E.S., Hankins C.A., MacHan J.T., Healey T.T., Dupuy D.E. (2013). Rib fractures after percutaneous radiofrequency and microwave ablation of lung tumors: incidence and relevance. Radiology.

[7] Hochberg U., Elgueta M.F., Perez J. (2017). Interventional analgesic management of lung cancer pain. Front Oncol.

[8] Arias J.I., Aller M.A., Arias J. (2009). Surgical inflammation: a pathophysiological rainbow. J Transl Med.

[9] Xu Q., Yaksh T.L. (2011). A brief comparison of the pathophysiology of inflammatory versus neuropathic pain. Curr Opin Anaesthesiol.

[10] Kehlet H., Jensen T.S., Woolf C.J. (2006). Persistent postsurgical pain: risk factors and prevention. Lancet.

[11] Jafri M.S. (2014). Mechanisms of myofascial pain. Int Sch Res Notices.

[12] Hopewell J.W. (2003). Radiation-therapy effects on bone density. Med Pediatr Oncol.

[13] Gerner P. (2008). Post thoracotomy pain management problems. Anesthesiol Clin.

[14] Chancellor W.Z., Mehaffey J.H., Desai R.P. (2021). Prolonged opioid use associated with reduced survival after lung cancer resection. Ann Thorac Surg.

[15] Maloney J., Wie C., Pew S. (2022). Post-thoracotomy pain syndrome. Curr Pain Headache Rep.

[16] Wildgaard K., Ravn J., Kehlet H. (2009). Chronic post-thoracotomy pain: a critical review of pathogenic mechanisms and strategies for prevention. Eur J Cardiothorac Surg.

[17] Bendixen M., Jørgensen O.D., Kronborg C., Andersen C., Licht P.B. (2016). Postoperative pain and quality of life after lobectomy via video-assisted thoracoscopic surgery or anterolateral thoracotomy for early stage lung cancer: a randomised controlled trial. Lancet Oncol.

[18] Long H., Lin Z.C., Lin Y.B., Situ D.R., Wang Y.N., Rong T.H. (2007). Quality of life after lobectomy for early stage non-small cell lung cancer--video-assisted thoracoscopic surgery versus minimal incision thoracotomy. Ai Zheng.

[19] Long H., Lin Z.C., Situ D.R. (2007). Cytokine responses after lobectomy: a prospective randomized comparison of video-assisted thoracoscopic surgery and minimal incision thoracotomy. Ai Zheng.

[20] Moghanaki D., Bravo Iñiguez C.E., Jaklitsch M.T., Extermann M. (2020). Geriatric Oncology.

[21] Kinsella T.J., Sindelar W.F., DeLuca A.M. (1985). Tolerance of peripheral nerve to intraoperative radiotherapy (IORT): clinical and experimental studies. Int J Radiat Oncol Biol Phys.

[22] Stoll B.A., Andrews J.T. (1966). Radiation-induced peripheral neuropathy. Br Med J.

[23] Rahi M.S., Parekh J., Pednekar P. (2021). Radiation-induced lung injury-current perspectives and management. Clin Pract.

[24] Gurewitz J., Mahadevan A., Cooper B.T. (2024). Pectoralis major radiation myonecrosis after lung stereotactic body radiation therapy. Pract Radiat Oncol.

[25] Nikitas J., DeWees T., Rehman S. (2019). Stereotactic body radiotherapy for early-stage multiple primary lung cancers. Clin Lung Cancer.

[26] Ball D., Mai G.T., Vinod S. (2019). Stereotactic ablative radiotherapy versus standard radiotherapy in stage 1 non-small-cell lung cancer (TROG 09.02 CHISEL): a phase 3, open-label, randomised controlled trial. Lancet Oncol.

[27] Voruganti I.S., Donovan E., Walker-Dilks C., Swaminath A. (2020). Chest wall toxicity after stereotactic radiation in early lung cancer: a systematic review. Curr Oncol.

[28] Kennedy W.R., Gabani P., Nikitas J. (2019). Treatment of T3N0 non-small cell lung cancer with chest wall invasion using stereotactic body radiotherapy. Clin Transl Radiat Oncol.

[29] Bongers E.M., Haasbeek C.J., Lagerwaard F.J., Slotman B.J., Senan S. (2011). Incidence and risk factors for chest wall toxicity after risk-adapted stereotactic radiotherapy for early-stage lung cancer. J Thorac Oncol.

[30] Carducci M.P., Sundaram B., Greenberger B.A., Werner-Wasik M., Kane G.C. (2023). Predictors and characteristics of Rib fracture following SBRT for lung tumors. BMC Cancer.

[31] Akhan O., Güler E., Akıncı D., Çiftçi T., Köse I.C. (2016). Radiofrequency ablation for lung tumors: outcomes, effects on survival, and prognostic factors. Diagn Interv Radiol.

[32] Palussière J., Chomy F., Savina M. (2018). Radiofrequency ablation of stage IA non-small cell lung cancer in patients ineligible for surgery: results of a prospective multicenter phase II trial. J Cardiothorac Surg.

[33] Hiraki T., Gobara H., Fujiwara H. (2013). Lung cancer ablation: complications. Semin Intervent Radiol.

[34] Moorjani N., Zhao F., Tian Y., Liang C., Kaluba J., Maiwand M.O. (2001). Effects of cryoanalgesia on post-thoracotomy pain and on the structure of intercostal nerves: a human prospective randomized trial and a histological study. Eur J Cardiothorac Surg.

[35] Chan V.O., McDermott S., Malone D.E., Dodd J.D. (2011). Percutaneous radiofrequency ablation of lung tumors: evaluation of the literature using evidence-based techniques. J Thorac Imaging.

[36] Wolf F.J., Grand D.J., Machan J.T., DiPetrillo T.A., Mayo-Smith W.W., Dupuy D.E. (2008). Microwave ablation of lung malignancies: effectiveness, CT findings, and safety in 50 patients. Radiology.

[37] Wang H., Littrup P.J., Duan Y., Zhang Y., Feng H., Nie Z. (2005). Thoracic masses treated with percutaneous cryotherapy: initial experience with more than 200 procedures. Radiology.

[38] Fain R., Thomas C.R., Nabavizadeh N. (2016). Shoulder pain after definitive lung radiotherapy. JAMA Oncol.

[39] Stephans K.L., Djemil T., Reddy C.A. (2009). A comparison of two stereotactic body radiation fractionation schedules for medically inoperable stage I non-small cell lung cancer: the Cleveland Clinic experience. J Thorac Oncol.

[40] Mesbah A., Yeung J., Gao F. (2016). Pain after thoracotomy. BJA Educ.

[41] Smith H.S. (2009). Potential analgesic mechanisms of acetaminophen. Pain Physician.

[42] Derry C.J., Derry S., Moore R.A. (2013). Single dose oral ibuprofen plus paracetamol (acetaminophen) for acute postoperative pain. Cochrane Database Syst Rev.

[43] Rainsford K.D. (2009). Ibuprofen: pharmacology, efficacy and safety. Inflammopharmacology.

[44] Pennefather S.H., Mckevith J. (2011). Principles and Practice of Anesthesia for Thoracic Surgery.

[45] Vyvey M. (2010). Steroids as pain relief adjuvants. Can Fam Physician.

[46] Watanabe S., Bruera E. (1994). Corticosteroids as adjuvant analgesics. J Pain Symptom Manage.

[47] Caplan A., Fett N., Rosenbach M., Werth V.P., Micheletti R.G. (2017). Prevention and management of glucocorticoid-induced side effects: a comprehensive review: Infectious complications and vaccination recommendations. J Am Acad Dermatol.

[48] Mathieson S., Lin C.C., Underwood M., Eldabe S. (2020). Pregabalin and gabapentin for pain. BMJ.

[49] Kukkar A., Bali A., Singh N., Jaggi A.S. (2013). Implications and mechanism of action of gabapentin in neuropathic pain. Arch Pharm Res.

[50] Sihoe A.D., Lee T.W., Wan I.Y., Thung K.H., Yim A.P. (2006). The use of gabapentin for post-operative and post-traumatic pain in thoracic surgery patients. Eur J Cardiothorac Surg.

[51] Finnerup N.B., Attal N., Haroutounian S. (2015). Pharmacotherapy for neuropathic pain in adults: a systematic review and meta-analysis. Lancet Neurol.

[52] Gül Ş.K., Tepetam H., Gül H.L. (2020). Duloxetine and pregabalin in neuropathic pain of lung cancer patients. Brain Behav.

[53] Trouvin A.P., Perrot S., Lloret-Linares C. (2017). Efficacy of venlafaxine in neuropathic pain: a narrative review of optimized treatment. Clin Ther.

[54] Aiyer R., Barkin R.L., Bhatia A. (2017). Treatment of neuropathic pain with venlafaxine: a systematic review. Pain Med.

[55] Kopsky D.J., Keppel Hesselink J.M., Bhaskar A., Hariton G., Romanenko V., Casale R. (2015). Analgesic effects of topical ketamine. Minerva Anestesiol.

[56] Wolff R.F., Bala M.M., Westwood M., Kessels A.G., Kleijnen J. (2010). 5% lidocaine medicated plaster in painful diabetic peripheral neuropathy (DPN): a systematic review. Swiss Med Wkly.

[57] Maloney J., Pew S., Wie C., Gupta R., Freeman J., Strand N. (2021). Comprehensive review of topical analgesics for chronic pain. Curr Pain Headache Rep.

[58] Mcnicol E.D., Midbari A., Eisenberg E. (2013). Opioids for neuropathic pain. Cochrane Database Syst Rev.

[59] DuPen A., Shen D., Ersek M. (2007). Mechanisms of opioid-induced tolerance and hyperalgesia. Pain Manag Nurs.

[60] Mercadante S., Arcuri E., Santoni A. (2019). Opioid-induced tolerance and hyperalgesia. CNS Drugs.

[61] Nicholls L., Gorayski P., Harvey J. (2015). Osteoradionecrosis of the ribs following breast radiotherapy. Case Rep Oncol.

[62] Pareek P., Samdariya S., Sharma A., Gupta N., Shekhar S., Kirubakaran R. (2016). Pentoxifylline and vitamin E alone or in combination for preventing and treating side effects of radiation therapy and concomitant chemoradiotherapy. Cochrane Database Syst Rev.

[63] Patil S., Sen S., Bral M. (2016). The role of acupuncture in pain management. Curr Pain Headache Rep.

[64] Vickers A.J., Vertosick E.A., Lewith G. (2018). Acupuncture for chronic pain: update of an individual patient data meta-analysis. J Pain.

[65] Roshanzamir S., Haririan Y., Ghaderpanah R., Jahromi L.S.M., Dabbaghmanesh A. (2023). Investigation of the effects of acupuncture on post-operative chest pain after open heart surgery. J Acupunct Meridian Stud.

[66] Urits I., Charipova K., Gress K. (2020). Treatment and management of myofascial pain syndrome. Best Pract Res Clin Anaesthesiol.

[67] Roldan C.J., Osuagwu U., Cardenas-Turanzas M., Huh B.K. (2020). Normal saline trigger point injections vs conventional active drug mix for myofascial pain syndromes. Am J Emerg Med.

[68] Hsu J., Yu S.P., Pan C.T., Huang P.M. (2023). Stripping massage and literature review in post-thoracoscopic chest pain management. Thorac Cardiovasc Surg.

[69] Ajimsha M.S., Al-Mudahka N.R., Al-Madzhar J.A. (2015). Effectiveness of myofascial release: systematic review of randomized controlled trials. J Bodyw Mov Ther.

[70] Kaplan J.A., Miller E.D., Gallagher E.G. (1975). Postoperative analgesia for thoracotomy patients. Anesth Analg.

[71] Takamori S., Yoshida S., Hayashi A., Matsuo T., Mitsuoka M., Shirouzu K. (2002). Intraoperative intercostal nerve blockade for postthoracotomy pain. Ann Thorac Surg.

[72] Choi E.J., Choi Y.M., Jang E.J., Kim J.Y., Kim T.K., Kim K.H. (2016). Neural ablation and regeneration in pain practice. Korean J Pain.

[73] Huang J., Delijani K., El Khudari H., Gunn A.J. (2022). Intercostal Cryoneurolysis. Semin Interv Radiol.

[74] Abd-Elsayed A., Lee S., Jackson M. (2018). Radiofrequency ablation for treating resistant intercostal neuralgia. Ochsner J.

[75] Moore W., Kolnick D., Tan J., Yu H.S. (2010). CT guided percutaneous cryoneurolysis for post thoracotomy pain syndrome: early experience and effectiveness. Acad Radiol.

